# Use of tailoring features and reasons for dropout in a guided internet-based transdiagnostic individually-tailored cognitive behavioral therapy for symptoms of depression and/or anxiety in college students

**DOI:** 10.1016/j.invent.2023.100646

**Published:** 2023-07-08

**Authors:** Marketa Ciharova, Pim Cuijpers, Yagmur Amanvermez, Heleen Riper, Anke M. Klein, Felix Bolinski, Leonore M. de Wit, Claudia M. van der Heijde, Ronny Bruffaerts, Sascha Struijs, Reinout W. Wiers, Eirini Karyotaki

**Affiliations:** a Department of Clinical, Neuro-, and Developmental Psychology, Vrije Universiteit Amsterdam, Van der Boechorststraat 7, BT 1081 Amsterdam, the Netherlands; b Amsterdam Public Health Research Institute, Vrije Universiteit Amsterdam, Van der Boechorststraat 7, BT 1081 Amsterdam, the Netherlands; c WHO Collaborating Center for Research and Dissemination of Psychological Interventions, Vrije Universiteit Amsterdam, Van der Boechorststraat 7, BT 1081 Amsterdam, the Netherlands; d Faculty of Medicine, Dept. of Psychiatry, University of Turku, FI-20014 Turun yliopisto, Finland; e Babeș-Bolyai University, International Institute for Psychotherapy, No.37, Republicii Street 400015, Cluj-Napoca, Romania; f Research Unit for Telepsychiatry and e-Mental Health, Department of Clinical Research, University of Southern Denmark, J.B. Winsløws Vej 19,3, 5000 Odense, Denmark; g Psychiatry, Amsterdam UMC, Vrije Universiteit Amsterdam, De Boelelaan 1118, 1081 HZ Amsterdam, the Netherlands; h Developmental and Educational Psychology of the Institute of Psychology, Leiden University, Rapenburg 70, 2311 EZ Leiden, the Netherlands; i Addiction, Development, and Psychopathology Lab, Department of Psychology, University of Amsterdam, Nieuwe Achtergracht 129, 1001NK Amsterdam, the Netherlands; j Department of Mental Health and Prevention, Trimbos Institute, Da Costakade 45, 3521 VS Utrecht, the Netherlands; k Department of Research, Development and Prevention, Student Health Service, University of Amsterdam, Oude Turfmarkt 151, 1012 GC Amsterdam, the Netherlands; l Universitair Psychiatrisch Centrum, Centre for Public Health Psychiatry, Katholieke Universiteit Leuven, Herestraat 49, 3000 Leuven, Belgium; m Center for Urban Mental Health, Oude Turfmarkt 145-147, 1012 GC Amsterdam, the Netherlands

**Keywords:** Adherence, Dropout, Digital intervention, College students, Transdiagnostic interventions, Individual tailoring

## Abstract

Transdiagnostic individually-tailored digital interventions reduce symptoms of depression and anxiety in adults with moderate effects. However, research into these approaches for college students is scarce and contradicting. In addition, the exact reasons for intervention dropout in this target group are not well known, and the use of individually-tailored intervention features, such as optional modules, has not yet been explored. The current study aimed to (1) investigate reasons for dropout from a guided internet-based transdiagnostic individually-tailored intervention for college students assessed in a randomized controlled trial (RCT) and (2) evaluate whether participants used tailoring features intended for their baseline symptoms. A sample of college students with mild to moderate depression and/or anxiety symptoms (*n* = 48) in the Netherlands (partially) followed a guided internet-based transdiagnostic individually-tailored intervention. We contacted those who did not complete the entire intervention (*n* = 29) by phone to report the reasons for intervention dropout. Further, we descriptively explored the use of tailoring features (i.e., depression versus anxiety trajectory) and optional modules of the intervention in the whole sample. We identified a range of person- and intervention-related reasons for intervention dropout, most commonly busy schedules, needs for different kinds of help, or absence of personal contact. Furthermore, only less than half of the participants used the individually-tailoring features to address the symptoms they reported as predominant. In conclusion, digital interventions clear about the content and targeted symptoms, tested in user research could prevent dropout and create reasonable expectations of the intervention. Participants would benefit from additional guidance when using tailoring features of digital interventions, as they often do not choose the tailoring features targeting their baseline symptoms.

## Highlights

• College students report person- and intervention-related reasons for dropout.
• Busy schedule or missing personal contact, among others, are reasons for dropout.
• College students using tailoring in digital interventions would favor from guidance.
• Digital interventions for college students should be understandable.
• User research should precede acceptability and effectiveness studies.

## 1 Introduction

College students are particularly vulnerable to depression and anxiety due to the developmental changes and a number of new stressors they face during this age period (Auerbach et al., 2018; Baghurst and Kelley, 2014). However, they often do not receive psychological treatment because of lack of time, preference for self-management of problems, stigma associated with the use of mental health care, and difficulty in finding the correct care (Czyz et al., 2013; Ebert et al., 2019; Farrer et al., 2011; Reichert, 2012). Digital interventions, i.e., those provided via information and communication technologies (ICT, such as the Internet), may be a promising way of alleviating the treatment gap in this population as they are highly accessible, cheap, acceptable, and less stigmatizing, thanks to their higher anonymity (Cuijpers et al., 2017; de Graaf et al., 2009). When administered with guidance from therapists or trained coaches, internet-delivered cognitive behavioral therapy (iCBT) has been found as effective as face-to-face interventions in reducing depressive and anxiety symptoms in adults, with moderate effect sizes when compared to inactive control conditions (Hedman-Lagerlöf et al., 2023; Karyotaki et al., 2018; Karyotaki et al., 2021; Pauley et al., 2021).

A promising way to optimize mental health interventions could be to address patients' individual needs using individually-tailored and transdiagnostic approaches. Tailoring entails adjustments to pieces of interventions based on the participants' unique clinical symptom presentation, personal preferences, and characteristics, such as motivation or education level (Păsărelu et al., 2017). Transdiagnostic approaches consist of interventions that concurrently address processes core to the progress and maintenance of more than one disorder (Craske, 2012). These approaches are particularly relevant for depression and anxiety due to their high comorbidities and a substantial overlap in symptoms, and etiologic factors (Auerbach et al., 2019; Garber and Weersing, 2010).

Transdiagnostic individually-tailored interventions reduce adult depression and anxiety with moderate effects, independent of the type of control group (i.e., active vs inactive; Newby et al., 2016; Păsărelu et al., 2017). However, the effects of digital interventions are much smaller in college students than in the general adult population and the dropout rate is considerable in this target group (Bolinski et al., 2020; Bolinski et al., 2022; Harrer et al., 2019). Furthermore, inconsistent findings were reported for anxiety and depression in trials comparing a transdiagnostic digital intervention to waitlist groups in college students, namely, no evidence of effects or large effects (Day et al., 2013; Mullin et al., 2015). Importantly, studies on individually-tailored interventions for this target group are lacking.

Against this, our recently conducted randomized controlled trial (RCT; Karyotaki et al., 2022) aimed to evaluate the effects of a guided internet-based transdiagnostic individually-tailored intervention “ICare Prevent”, built on the best evidence to create digital interventions adapted for college students (Furukawa et al., 2021), in comparison to treatment as usual in college students (TAU). However, we found no evidence of a difference between ICare Prevent and TAU in any of the examined outcomes (i.e., depressive and anxiety symptom severity, quality of life, educational achievement), and 46 % of participants dropped out before completing the core modules of the intervention. Potential reasons for the absence of significant differences among the examined groups included: low severity of the participants' complaints at baseline, recruitment of participants who did not actively seek help, and insufficient statistical power to detect a small but clinically meaningful effect size, among others. Although we did not find any association between dropout from the intervention and intervention effectiveness, we could not rule out the possibility of an association due to limited statistical power.

Dropout from digital interventions may be related to intervention outcomes, as it does not occur by chance, and thus, specific subgroups of participants may receive insufficient exposure to the intervention (Donkin et al., 2011; Karyotaki et al., 2015). Developing and tailoring transdiagnostic digital interventions would benefit greatly from identifying individuals more likely to drop out and the exact reasons for their dropout. Differing expectations, improvement or worsening of health, preference for face-to-face CBT, negative perceptions about iCBT, low motivation, lack of time, internet delivery problems, and unexpected personal circumstances were reported as reasons for dropout in general populations, while the last three have also been reported in college students (Børtveit et al., 2022; Kaltenthaler et al., 2008; Marks et al., 2003; Melville et al., 2010; Noone and Hogan, 2018; Rost et al., 2017; Schneider et al., 2014; Treanor et al., 2021; Waller and Gilbody, 2009). However, reasons for intervention dropout have been severely under-investigated in college students whose digital literacy may be higher and who may differ in lifestyle from general adult populations and thus in reasons for dropout (Abdulai et al., 2021; Ferrari et al., 2022; Moreno-Gómez et al., 2012). It is also largely unknown whether reasons for dropout diverge among participants at different intervention stages (Karyotaki et al., 2015; van Ballegooijen et al., 2014).

One possible way to reduce dropout is tailoring the intervention to the specific participant (Pearl and Norton, 2017). However, studies on individually-tailoring elements in digital interventions and their effectiveness are also scarce. It has to be clarified whether participants know how to take advantage of the individually-tailoring features to adjust the intervention to suit their needs (Mukhiya et al., 2020).

Improving adherence to digital interventions for college students is crucial for their sustainable implementation in university settings (Lattie et al., 2019). Studies exploring 1) the reasons for dropout and 2) use of transdiagnostic individually-tailored interventions for college students are thus warranted. Thus, in the current study, we aimed to (1) investigate reasons for dropout from “ICare Prevent” and assess whether they differ for dropout at different stages of the intervention and (2) evaluate whether participants use tailoring features intended for their baseline symptoms. We hypothesized that the reasons for dropout would overlap with those found in adult populations but would not include negative perceptions of digital interventions. In addition, we expected that the participants would use tailoring features of the intervention in line with their symptoms reported at baseline: (1) they would follow either a depression or anxiety trajectory, according to the symptoms they experienced, and (2) they would complete the optional modules meant to address other complaints they had previously reported.

## 2 Methods

### 2.1 Design

This study is part of the World Mental Health International College Student Initiative (WHO WMH-ICS; Cuijpers et al., 2019). Data analyzed here come from an RCT evaluating the effectiveness of a guided transdiagnostic individually-tailored internet-based cognitive behavioral therapy addressing depressive and anxiety symptoms in college students. For a detailed description of the methods, measures, results and ethical considerations of the RCT on which we conducted a secondary analysis, the reader can refer to the protocol and the primary publication (Karyotaki et al., 2019; Karyotaki et al., 2022).

In the current study, we only analyzed data from the screening, baseline and post-treatment of the intervention group. We conducted qualitative analysis, using interview data, to examine reasons for dropout from the intervention. Quantitative analysis evaluated the association between reasons for dropout and number of completed sessions, and the frequency of use of tailoring features in relation with participants' symptoms.

### 2.2 Participants and procedure

Researchers from the Vrije Universiteit Amsterdam (VU) and the University of Amsterdam (UvA) in the Netherlands conducted this study from March 2018 through June 2020. Participants were recruited via printed advertisements, word-of-mouth and automatized e-mails in their mailboxes and asked to complete a screening survey covering a range of topics related to mental health (Cuijpers et al., 2021). They were eligible for the RCT if they (1) were students at a university in the Netherlands; (2) were at least 18 years old; (3) were fluent in Dutch or English (self-reported); (4) reported mild to moderate depression and/or anxiety symptoms defined as cut-off scores above four on the Patient Health Questionnaire (PHQ-9; Kroenke et al., 2001) and the Generalized Anxiety Disorder scale (GAD-7; Spitzer et al., 2006), respectively; and (5) provided written informed consent prior to participation in the trial. Exclusion criteria were: (1) bipolar disorder (based on a telephone-delivered MINI International Neuropsychiatric Interview; MINI; Sheehan et al., 1998); (2) severe depressive and/or anxiety symptoms (i.e., scoring above the scores of 14 on PHQ-9 and GAD-7, respectively); (3) reception of psychological treatment for depression and/or anxiety in the past 12 months; and (4) slow or no internet connection (self-report).

### 2.3 Intervention

In the current study, we describe the individually-tailoring features and optional modules in more detail (see the protocol for more information about the intervention, Karyotaki et al., 2019). The intervention was sequential (i.e., a participant had to follow the core sessions in a pre-specified order) and tailored in two ways: (1) Participants decided whether they preferred to focus on symptoms of depression (problem-solving) or anxiety (exposure in daily life) in the fifth and sixth session (out of total seven). All participants chose only one trajectory, which they subsequently followed in sessions 5 and 6. (2) Optional modules could be followed in sessions 2–7. These modules were either general (beneficial for coping with any complaints, i.e., acceptance of unfulfilled needs, relaxation, reducing brooding, and appreciation and gratitude) or specific (addressing specific difficulties, i.e., alcohol consumption as emotion regulator, sleep hygiene, perfectionism, worries, and self-worth; Appendix A). Each participant could follow only one optional module in every session, but also each more than once. Even if a participant had already completed an optional module, the intervention would continue to offer this module automatically in the subsequent sessions.

If a participant did not complete a session after completing the previous one, they received a reminder message on the platform. In addition, they also received an email notification informing them that they had a new message on the platform.

### 2.4 Measures

#### 2.4.1 Adherence per protocol

Adherence was a dichotomous variable depending on whether the participant completed the intervention as per protocol, which required completion of four out of seven sessions, and thus followed both the core components of iCBT (i.e., behavioral activation and cognitive restructuring). We calculated the number of completed sessions according to the log-files of the digital intervention.

#### 2.4.2 Depressive symptoms

To assess depressive symptoms, we used the PHQ-9 (Kroenke & Spitzer, 2002).

#### 2.4.3 Anxiety symptoms

To examine anxiety, we used the GAD-7 (Spitzer et al., 2006).

#### 2.4.4 Current diagnosis of major depressive episode (MDE) and generalized anxiety disorder (GAD), panic disorder, agoraphobia and social phobia

The MINI, a brief structured diagnostic interview (Sheehan et al., 1998), was used to establish current diagnoses of MDE and panic disorder, and current GAD, agoraphobia and social phobia.

#### 2.4.5 Reasons for dropout from intervention

Participants who did not follow all seven sessions of the intervention were contacted via telephone and asked why they did not complete the intervention.

#### 2.4.6 Choosing between problem-solving versus exposure module

Although we recruited participants based on both depression and anxiety symptoms, only some participants were diagnosed with current MDE or an anxiety disorder (MINI). Moreover, all participants reported a higher symptom severity in one of the two disorders, suggesting that either depression or anxiety symptoms were predominant in each participant. Thus, we categorised the participants into two groups, according to which symptoms they reported predominantly (i.e., a group with predominant depression or anxiety symptoms) and examined whether, in sessions 5 and 6, participants chose the trajectory addressing their predominant symptoms (i.e., problem-solving for depressive and exposure for anxiety symptoms). The decision about whether depression or anxiety symptoms were predominant in each participant was made based on the following hierarchy:

1. Disorder diagnosis (i.e., MDE or any anxiety disorder based on the MINI);
2. If a participant had both depression and anxiety diagnoses, or no diagnosis, the decision was made according to the symptoms of which they had a higher severity (i.e., mild or moderate severity based on PHQ-9 or GAD-7);
3. If the participant had equal symptom severity for both depression and anxiety, the decision was made according to symptoms for which they were included in the trial (i.e., total score > 4 on PHQ-9 or GAD-7);
4. If the participant met both the cut-offs, the score on PHQ-9 and GAD-7 was considered.

We conducted sensitivity analyses when defining the predominance of depressive or anxiety symptoms only based on (i) the symptoms relevant to the inclusion of the participant, (ii) higher severity of symptoms, and (iii) the diagnosis.

#### 2.4.7 Choosing an optional module matching to current emotional problems

We investigated whether participants followed optional modules and, if they did, whether these modules were either general or specific in nature. If the participant followed specific modules, we examined whether these modules addressed the psychological problems the participants reported during the screening. These problems involved hazardous alcohol consumption, insomnia, worries, perfectionism, and low self-esteem. We assessed hazardous alcohol consumption with the 10-item version of the Alcohol Use Disorders Identification Test (AUDIT; Saunders et al., 1993), which has good concordance with clinical diagnoses (AUC of 0.78–0.91; Reinert & Allen, 2007), and where hazardous consumption is a total score of ≥8. We measured the presence of insomnia with the 7-item Insomnia Severity Index (ISI), a validated and reliable self-report measure, where having (subthreshold) insomnia is indicated by a score of 8 or higher (Bastien et al., 2001). The presence of worrying, perfectionism and low self-esteem was evaluated by asking the participants how much they agreed with the following statements on a 5-point Likert scale, ranging from 1 (“Never”) to 5 (“All the time”): “You are worrying too often and you have trouble getting rid of these thoughts”, “You have too much urge to do everything perfectly”, and “You do not value or appreciate yourself”, respectively. We dichotomised the answers to “Yes” and “No”, while the cut-off for “Yes” was set at 3 (“Regularly”; Nansel et al., 2001). We then determined whether participants who reported these problems effectively followed the modules designed to address them, i.e., alcohol consumption as an emotion regulator, sleep hygiene, worries, perfectionism, and self-worth. If a participant followed a module addressing a problem they reported, we considered this module to match their problems (hereafter referred to as: “matched module”). We further looked into the potential reasons for not following matched modules. The reasons could be: 1) a participant dropped out from the intervention before reaching the session where the first optional module was introduced (i.e., the second session), 2) a participant chose not to complete any optional module, 3) a participant completed another matched optional module or a general optional module instead (i.e., acceptance of unfulfilled needs, relaxation, reducing brooding, and appreciation and gratitude), or 4) a participant chose an optional module which addressed a problem they did not suffer from (hereafter referred to as: “unmatched module”).

### 2.5 Analysis

We performed the analyses in Stata/SE, version 16.1 for Mac (StataCorp., 2019). We used frequency statistics to describe the sample in terms of demographic characteristics, numbers of completed sessions, optional modules, chosen (depression or anxiety) trajectory and reasons for intervention dropout.

Two independent researchers (MC and YA) applied grounded theory methodology to the answers to the question of why participants did not complete the intervention. First, both independently open-coded the answers to identify concepts and key phrases in each answer. Then they sorted the codes into subcategories, labelled the relationships between these subcategories (axial coding) and classified these subcategories into core categories (selective coding; Noble and Mitchell, 2016). They compared their coding and resolved any discrepancies through discussion with a senior researcher (EK).

We used penalized logistic regression for rare events to compare whether any reasons for dropout were associated with the number of completed sessions among participants who dropped out (Firth, 1993; King and Zeng, 2001). In addition, we used χ^2^-test or Fisher's exact test (if an expected count in >20 % of cells was lower than 5; Field, 2013) to assess whether participants who reported a problem (i.e., alcohol consumption, insomnia, worry, perfectionism, low self-esteem) were more likely to complete an optional module addressing this problem than participants who did not report this problem. Similarly, we examined whether participants reporting predominant depressive symptoms were more likely to choose the problem-solving module than participants reporting predominant anxiety symptoms, and whether participants reporting predominant anxiety symptoms were more likely to choose the exposure module.

## 3 Results

### 3.1 Descriptive analyses

The average age of the intervention group was 21.75 years (*SD* = 2.70), and 38 of the 48 participants were female (82.7 %). Most participants (*n* = 35; 72.9 %) attended VU Amsterdam and were bachelor students (*n* = 31; 64.6 %) of Dutch nationality (*n* = 35; 72.9 %) and ethnicity (*n* = 30; 62.5 %). Most participants (*n* = 35; 72.9 %) were invited to the trial due to mild to moderate symptoms of both depression and anxiety, while respectively, 9 (18.8 %) and 4 (8.3 %) participants were invited for depressive and anxiety symptoms only.

The mean number of completed sessions was 4.50 (*SD* = 3.04) out of seven, and the mean number of optional modules was 2.67 (*SD* = 2.14). Twenty-six participants (54.2 %) completed the intervention as per protocol, while 19 (39.6 %) completed all seven sessions, 15 (31 %) reached the optional booster session (session 8), and 36 participants (75 %) followed at least one optional module. The steepest dropout of participants (*n* = 8; 16.7 %) took place after the third session, while dropout was milder after this point, e.g., only one participant dropped out after the sixth session (Appendix B).

### 3.2 Reasons for intervention dropout

Out of the 29 participants who did not complete at least seven sessions (i.e., the entire intervention), 24 (82.8 %) answered the question about their dropout reason(s). The final thematic framework covers two general groups of topics: (1) the participant's individual characteristics and (2) the intervention's format and content (Table 1).

**Table 1:** Final thematic framework for reasons for dropout.

Topic	Reason	Example of quotation
Person-related	Too busy schedule	“I did not participate in the intervention because of preparations for my trip and thesis. It seemed like it would take a lot of time.”
	Major life changes	“I was at the beginning of my second year. I had a lot of things going on then: I moved about 3 or 4 times, I had stress about my study, (…), I had to start socializing to make new friends.”
	Mild symptoms	“I was not really motivated to do it, I really liked the modules, but I felt that I did not have any complaints and the intervention was not needed for me.”
	Improvement in symptoms	“My reason for stopping sooner was that I noticed that I was feeling very well mentally quite soon. Caring Universities contributed to this.”
	Need for different help	“The situation got worse, so I went to a psychologist. The intervention was a good first step, then was not helping anymore.”
	Impact of depressive symptoms	“When a person is already tired and depressed, it is hard to concentrate for that long and get to do something like that regularly.”
	Perfectionism	“Participating in the first assignment felt like a lot of hard work and like a performance or achievement at which I had to be really good.”
Intervention-related	Lack of personal contact	“I did not feel motivated, because I had problems and felt I wanted a different support. I missed the personal contact.”
	Lack of pressure to follow the intervention	“The problem was that nothing was making me follow the intervention, there was no punishment if I did not do it, no-one was waiting for me at the exact time and place, no-one was controlling if I really did it, I paid no money for it.”
	Preference of speaking over writing	“It was hard for me to write things down, instead of speaking it out to someone else.”
	Too basic/superficial	“I think I had very high expectations and was disappointed by the first assignment. It felt like scratching the surface and trying to think of practical tools for handling yourself, and I was answering the questions with what I thought were the right answers but I felt like it didn't look further or at the core of the problem.”
	Perceived lack of effectiveness	“The reason in the end was that I had the feeling it did not really help me.”
	Previous experience with a similar intervention	“I opened it and felt like the intervention was one of those I did as a child. (…) There was always a child presented with allegedly the same problem, for example, the child was afraid of the same thing. However, it was not real and it did not help me.”
	Lack of access to the internet	“(I joined) at the beginning, but then I went on an unplanned trip, so I couldn't really follow the intervention because of bad internet abroad.”

The most common reason for dropout, reported by 15 participants (Table 2), was that they were too busy, and one participant reported major life changes during the intervention. Nine participants reported that their needs differed from what they received in the intervention. For example, two participants reported that their symptoms were too mild in the first place, the symptoms of one participant improved during the study, and six sought a different type of help instead. Moreover, two participants had problems with the planning of the intervention and with concentration, which they attributed to the depressive symptoms, and one with perfectionist traits, which made the intervention seem too demanding to them.

**Table 2:** Association between the number of completed sessions and (providing) reasons for dropout.

	n (%)	Mean Number of Sessions (*SE*)	OR (95 % CI)	
	29 (100)	2.34 (0.33)		p
Provided a reason	24 (82.8)	2.54 (0.37)	1.41 (0.79, 2.50)	0.245
Provided reasons				
Person-related				
Too busy schedule	15 (51.7)	2.60 (0.47)	1.04 (0.67, 1.62)	0.847
Major life changes	1 (3.5)	1 (NA)	0.72 (0.26, 1.95)	0.512
Mild symptoms	2 (6.9)	2 (2)	0.87 (0.42, 1.79)	0.707
Improvement in symptoms	1 (3.5)	5 (NA)	1.89 (0.53, 6.66)	0.324
Need for different help	6 (20.7)	2.17 (0.75)	0.87 (0.53, 1.43)	0.584
Impact of depressive symptoms	2 (6.9)	2.50 (0.5)	0.99 (0.49, 2.01)	0.984
Perfectionism	1 (3.5)	1 (NA)	0.72 (0.26, 1.95)	0.512
Intervention-related				
Lack of personal contact	5 (17.2)	1.60 (0.51)	0.71 (0.40, 1.25)	0.237
Lack of pressure to follow the intervention	5 (17.2)	3.60 (0.81)	1.48 (0.83, 2.63)	0.187
Preference of speaking over writing	1 (3.5)	1 (NA)	0.72 (0.26, 1.95)	0.512
Too basic/superficial	3 (10.3)	2 (0.58)	0.85 (0.46, 1.60)	0.622
Perceived lack of effectiveness	5 (17.2)	2.2 (0.8)	0.89 (0.53, 1.50)	0.661
Previous experience with a similar intervention	3 (10.3)	0.7 (0.34)	0.49 (0.20, 1.21)	0.122
Lack of access to the internet	1 (3.5)	0 (NA)	0.51 (0.13, 1.96)	0.326

*Note.* Abbreviations (alphabetical): *95* *% CI*: Confidence interval; NA: Not available (in case of only 1 participant); *OR*: Odds ratio; *p*: *p*-value.

Regarding the intervention-related reasons, participants sometimes said they would prefer more personal contact (*n* = 5). Five participants also mentioned that there was no pressure to complete subsequent sessions, and one would have preferred to express their thoughts in speaking instead of writing. The intervention felt too basic or superficial to three participants, and five thought that it did not really help their problems. Three participants had previous experience with a similar intervention, but since the previous intervention had not helped them, they were not motivated to follow it again. One participant could not follow the intervention because they had unexpectedly no access to the internet due to a change of plans including travel to a country with a slow internet connection. No association was found between the number of completed sessions and providing the reason for dropout (*p* > .05), neither was there an association between the number of completed sessions and reporting any of the reasons mentioned above (all *p* > .05), suggesting no evidence of a difference between reasons for dropout at different stages of the intervention.

Some participants also spontaneously mentioned that they liked the intervention (*n* = 4), found it useful (*n* = 3) or a good first step towards tackling their mental health complaints (*n* = 1). Participants specifically appreciated practical tips (e.g., relaxation and breathing exercises; *n* = 1) and optional modules (*n* = 1). Despite their dropout, two participants liked the intervention more than visiting a psychotherapist, as they could follow the sessions whenever it suited their schedule and energy. One participant suggested that it would help them if the intervention followed a smartphone app, where they could receive reminders and messages from the coach. Reminders often fell into the spam folder in the participant's inbox, and even if the participant read this notification, the automated message only informed them that they should visit the website and read the message from the coach. However, the content of the message was not included (for legal reasons). Visiting the website thus represented an additional burden in reading the reminder mentioned by two participants. One participant also wished to be more connected to the coach via the messaging feature, beyond sole feedback to sessions and reminders.

### 3.3 Tailoring features – depression and anxiety trajectory

Twenty-three participants completed at least five sessions and thus had to choose between problem-solving and exposure modules as part of the individually-tailoring feature. Among these participants, 14 (60.9 %) reported depressive and nine (39.1 %) anxiety symptoms as predominant (based on the hierarchy of measures as explained above), respectively. However, only 10 participants (43.4 %) chose the module specialised in symptoms that they reported as predominant. Participants with predominant depressive symptoms were not more likely to complete the problem-solving module than participants with predominant anxiety symptoms. Concurrently, participants with predominant anxiety symptoms were not more likely than participants with predominant depressive symptoms to complete the exposure module (*p* > .05). The results of the sensitivity analyses, where we used different definitions of predominant symptoms were used, are in Appendix C.

### 3.4 Tailoring features – optional modules

Twelve participants did not follow any optional module, while seven completed the maximum number of optional modules (*n* = 6; Appendix D). The most popular optional module was self-worth, followed by 23 participants (47.9 %; Table 3), while the least popular module was alcohol consumption (*n* = 5; 10.4 %). Between 17.4 % (*n* = 4; alcohol consumption) and 55.9 % (*n* = 19; self-worth) of participants completed a module addressing a problem they reported.

**Table 3:** Number of participants completing problem-solving, exposure and optional modules.

	48 (100)	Reported the problem^a^		With vs without the problem^b^	
				χ^2^	p
Problem-solving vs exposure module		All participants *N* = 48	N sessions ≥ 5 *N* = 23	d	0.999
Problem-solving		29 (100)	14 (100)^c^		
Completed	9 (18.8)	5 (17.2)	5 (35.7)		
Did not complete	39 (81.2)	24 (82.8)	9 (64.3)		
Exposure		19 (100)	9 (100)^c^	e	
Completed	14 (29.2)	5 (26.3)	5 (55.6)		
Did not complete	34 (70.83)	14 (73.7)	4 (44.4)		
General modules		All participants *N* = 48	N sessions ≥ 2 *N* = 38		
Acceptance of unfulfilled needs	11 (22.9)	f	11 (22.9)	f	f
Relaxation	18 (37.5)	f	18 (37.5)	f	f
Reducing brooding	9 (18.75)	f	9 (18.75)	f	f
Appreciation and gratitude	11 (2.9)	f	11 (2.9)	f	f
Specific modules					
Alcohol consumption as emotion regulator		23 (100)^c^	16 (100)^c^	d	0.141
Completed	5 (10.4)	4 (17.4)	4 (25.0)		
Did not complete	43 (89.6)	19 (82.6)	12 (75.0)		
Sleep hygiene		29 (100)^c^	21 (100)^c^	3.98	0.046^⁎^
Completed	18 (37.5)	13 (44.8)	13 (61.9)		
Did not complete	30 (62.5)	16 (55.2)	8 (38.1)		
Worry		40 (100)^c^	31 (100)^c^	d	0.034^⁎^
Completed	13 (27.1)	8 (20)	8 (25.8)		
Did not complete	35 (72.9)	32 (80)	23 (74.2)		
Perfectionism		29 (100)^c^	25 (100)^c^	d	0.077
Completed	14 (29.2)	12 (41.4)	12 (48.0)		
Did not complete	34 (70.8)	17 (58.6)	13 (52.0)		
Self-worth		34 (100)^c^	27 (100)^c^	d	0.073
Completed	23 (47.9)	19 (55.9)	19 (70.4)		
Did not complete	25 (52.1)	15 (44.1)	8 (28.6)		

Note.

a Whether in the screening survey (T0) the participant reported suffering from the problem for which the given specific module is meant, meaning that the participant suffered predominately from depressive or anxiety symptoms (modules: problem-solving or exposure, respectively), that they scored 8 or higher on AUDIT (matched module: alcohol consumption as emotion regulator) or ISI (matched module: sleep hygiene), or they reported suffering from worrying, perfectionism or low self-esteem (matched modules: worries, perfectionism, self-worth, respectively).

b Comparison whether participants who reported the problem addressed by the given module were more likely to complete the given module than participants who did not report the problem.

c Percentage calculated from the total number of participants (100 %) who reported the problem.

d Fisher's test calculated instead of χ^2^-test (expected counts were <5 for >20 % of cells).

e Since participants who did not complete the problem-solving module completed all the exposure module, additional statistical test is redundant.

f Missing data – as the module is general, there was no specific measure to assess whether the participant suffered from the problem which the modules were addressing.

Participants completed, on average, 1.17 (*SE* = 0.15) matched modules, 1.02 (*SE* = 0.17) general modules, and 0.35 (*SE* = 0.08) unmatched modules. After the intervention, on average, 2.06 (*SE* = 0.19) problems previously reported remained unaddressed by an optional module (from the total of 3.22 problems previously reported, *SE* = 0.19). Appendix E shows a detailed grouping of participants according to whether they did or did not address all their problems via optional modules. Among those who did not address all their problems (*n* = 42; 87.5 %), 16 (33.4 %) participants did not complete all the optional modules needed because they either dropped out before such an opportunity or because they chose not to complete a sufficient number of optional modules. The rest, i.e., 26 (54.2 %) participants, completed at least one general or at least one unmatched instead of matched module. In addition, the participants who addressed all their problems (6; 12.5 %) also completed a mean of 1.17 (*SE* = 0.65) general and 0.83 (*SE* = 0.17) unmatched modules besides the matched modules.

Some participants completed the same module multiple times. Four participants completed the same module (Worries) twice or three times, and two out of these participants did not report suffering from worrying. Moreover, one participant completed the general module reducing brooding twice.

In a subsample of participants, who completed at least two sessions and were thus able to follow an optional module, there was no evidence of any difference between participants who reported hazardous alcohol consumption, perfectionism and low self-esteem, respectively, and those who did not, in completing the respective optional modules. Participants who reported insomnia (*n* = 13/21; 61.9 %) were more likely to complete the sleep hygiene module than participants who did not report insomnia (*n* = 5/17; 29.4 %; *p* = .046). However, participants who reported suffering from worrying (*n* = 8/31; 25.8 %) were less likely than participants who did not report worries (*n* = 5/7; 71.4 %; *p* = .034) to complete the module on worries.

## 4 Discussion

The current study aimed to (1) explore reasons for dropout from a guided internet-based transdiagnostic individually-tailored intervention and assess whether they differ for dropout at different stages of the intervention and (2) evaluate whether participants used tailoring features intended for them. We identified various person- and intervention-related reasons for intervention dropout, such as a busy schedule, need for a different kind of help or absence of personal contact. There was no association between the type of reason and the intervention stage in which the participant dropped out. In addition, less than half of the participants chose optional modules aimed at the symptoms they reported.

Several identified reasons for intervention dropout are coherent with findings in the general population and college students, such as being too busy to complete the intervention (Børtveit et al., 2022; Kaltenthaler et al., 2008; Marks et al., 2003; Melville et al., 2010; Noone and Hogan, 2018; Rost et al., 2017; Schneider et al., 2014; Treanor et al., 2021; Waller and Gilbody, 2009). Participants in the current study did not explicitly mention such preferences. However, some reasons suggest they may prefer face-to-face over digital interventions: They missed personal contact, preferred to express thoughts in speaking, needed a different kind of help (e.g., face-to-face psychotherapy), and felt no pressure to follow the intervention (i.e., no fee or scheduled appointments). This finding is surprising considering the potential association between higher digital literacy and preferences for digital contact in this target group (Abdulai et al., 2021).

Some participants considered the intervention too basic or superficial. While this may be specific to college students or a problem of the current intervention, the perception of face-to-face CBT as manualised, symptom-focused, and surface-level has already been reported (Binnie, 2012). None of the participants reported negative perceptions about the digital nature of the intervention in particular: Therefore, the opinions identified in the present study may not be necessarily limited to iCBT. Nevertheless, coaching had a minimal extent, only ensuring the completion of sessions. The participants had limited interaction with coaches and never reflected on their feedback or the intervention content. The lack of contact may have created disappointment, as there was no space to discuss how the content related to participants' symptoms, leading to the perception of the intervention as superficial. In contrast, participants may have more room for reflection with the therapist or coach in synchronous feedback, such as a face-to-face or phone-supported intervention. For example, phone support relates to higher adherence than e-mail support (Wojtowicz et al., 2013).

Fourteen participants dropped out because the current intervention reminded them of a digital intervention they had previously followed and not enjoyed, they did not perceive the intervention as effective, or they needed a different kind of help. Dropout is higher in participants perceiving an intervention as unhelpful, uncredible, inconvenient, or not meeting their preferences and needs (Beatty and Binnion, 2016; Kaltenthaler et al., 2008; Rost et al., 2017; Vallury et al., 2015). Thus, participants may make conclusions about the effectiveness of an intervention based on their past experiences.

Less than half of the study participants chose matched modules. Even if a participant reported a problem for which individually-tailoring features were designed, with the exception of sleep hygiene, the participant was not more likely to complete this module than participants who did not report the problem. Participants who reported worrying were less likely to follow the matched module than others. This result is coherent with previous findings that students choose an intervention based on the symptoms they consider the most urgent, even when it is inconsistent with results from self-report instruments (Palacios et al., 2018). While even unmatched modules may lead to improvement, it is possible that some participants are not fully aware of their needs. For example, college students use alcohol consumption module very little (Bolinski et al., 2022), suggesting that they do not perceive alcohol use as a problem. Unclear formulations of the content and symptoms targeted by the modules may also cause unmatched decisions. Before participants decided to follow the problem-solving or exposure module, they received descriptions of what to expect from them. Although these descriptions listed the symptoms of depression and anxiety, respectively, it is possible that participants could not recognize their own symptoms in such descriptions.

This study adds to the under-researched topic of reasons for intervention dropout, and the use of tailoring features in digital interventions for college students, thus providing insights for developing future interventions. However, certain limitations must be acknowledged. We did not ask the participants specifically about the advantages of the investigated intervention, tips for improvement, or reflection on their choices. We thus only report what participants spontaneously mentioned while being asked about reasons for dropout. Future research should explore suggestions for improvement. Furthermore, most participants reported symptoms of both depression and anxiety. It is hence difficult to distinguish which symptoms were predominant and whether participants followed the correct (problem-solving or exposure) trajectory. However, we based the main distinction on diagnoses of the clinician-rated MINI, and all sensitivity analyses revealed the same results. Finally, due to low statistical power, generalizability of the results is limited.

There may be a connection between the current findings and the lack of effects observed in our effectiveness study (Karyotaki et al., 2022). Regarding the discovered reasons for dropout, the length of sessions, colliding with busy schedules, might have caused participants to only skim through the content. This causes poor engagement with the intervention and subsequently contributes to the insufficient implementation of newly acquired knowledge into daily lives (Kelders, 2019). In addition, we need to explore whether participants perceive synchronous feedback as addressing the core of their problems better than asynchronous feedback. Moreover, future research should determine whether brief interventions better suit college students' busy schedules and thus (partially) prevent dropout. Authors of future digital interventions should also use a web-app environment to enable participants to receive reminders to complete sessions and messages from coaches.

Considering the findings related to tailoring features, participants might have felt overwhelmed by the decisions they had to make, leading them to decisions that did not reflect their needs. Therefore, users of digital interventions would benefit from additional help when choosing optional modules according to baseline symptoms. Furthermore, explaining as transparently as possible what symptoms optional modules address is important. These formulations should be tested in user research before any acceptability or effectiveness assessment to investigate what expectations such explanations trigger. If participants select an optional module they have previously followed, it is important to alert them about their choice before proceeding. Finally, baseline examinations of participants' experiences with similar treatments could inform tailored interventions reflecting these experiences.

In conclusion, college students report a range of person- and intervention-related reasons for dropout from guided internet-based transdiagnostic individually-tailored interventions. These include a busy schedule, the need for different kinds of help, or the absence of personal contact. Interventions with clear explanations of the content and targeted symptoms of the modules could partially prevent dropout. User research testing expectations about the intervention should precede any acceptability or effectiveness studies. Participants would benefit from additional help when using tailoring features so that these address their baseline symptoms.

## Funding

This trial was supported by 10.13039/501100001826ZonMw, Research Program GGz [grant number 636110005].

## Declaration of competing interest

The authors declare that they have no known competing financial interests or personal relationships that could have appeared to influence the work reported in this paper.

## Figures and Tables

**Figure:**
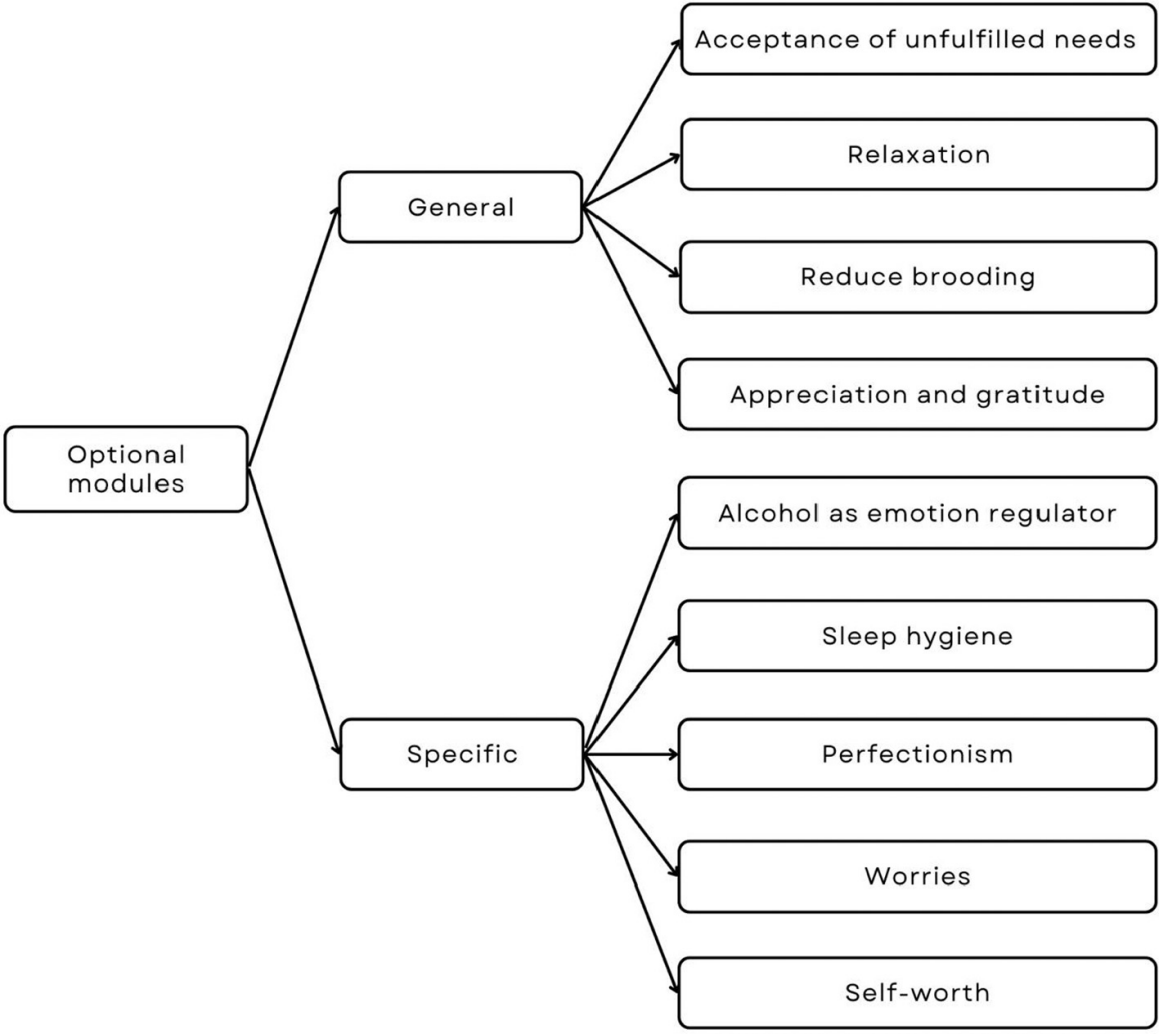


**Table:** 

Number of participants completing each number of sessions	
	*N (%)*
0	7 (14.6)
1	3 (6.3)
2	4 (8.3)
3	8 (16.7)
4	3 (6.3)
5	3 (6.3)
6	1 (2.1)
7	4 (8.3)
8 (7 + booster)	15 (31.3)

**Table:** 

Number of participants completing the matched individually tailored trajectory^a^		
Predominant^b^	*N* (%)	Correct module
Inclusion^c^	**23 (100)**^g^	**11 (47.8)**
Depressive symptoms	5 (21.7)	1 (4.4)
Anxiety symptoms	1 (4.4)	0 (0.0)
Both^d^	17 (73.9)	10 (43.4)
Severity^e^	**23 (100)**^g^	**11 (47.8)**
Depressive symptoms	13 (56.5)	5 (21.7)
Anxiety symptoms	2 (8.7)	1 (4.4)
Both^d^	8 (34.8)	5 (21.7)
Diagnoses^f^	**23 (100)**^g^	**10 (43.5)**
Depression	2 (8.7)	1 (4.4)
Anxiety	6 (26.1)	3 (13.0)
Both or none^d^	15 (65.2)	6 (26.1)

*Note*.

a Matched individually tailored trajectory refers to completing the problem-solving module if the participant had more severe depressive than anxiety symptoms or a diagnosis of depression, but not anxiety, or completing the exposure module if a participant had more severe anxiety than depressive symptoms or a diagnosis of anxiety, but not depression.

b The symptoms of the disorder which the participant scored higher on or diagnoses the participant had.

c The symptoms for which the participant was included (total score > 4 on PHQ-9 or GAD-7).

d If a participant was included for both disorders, had the same severity of both disorders or had the diagnosis of both or none of the disorders, matched trajectory was considered the module aimed at the disorder with higher total score on PHQ-9 or GAD-7. If the score was exactly the same, any trajectory the participant chose was considered a matched trajectory.

e The disorder of which the participant had a higher severity (based on PHQ-9 and GAD-7).

f The diagnosis the participant had (based on MINI).

g Only participants who completed at least 5 sessions are considered.

**Table:** 

Number of participants completing each number of optional modules	
	*N* (%)
0	12 (25)
1	3 (6.3)
2	11 (22.9)
3	4 (8.3)
4	6 (12.5)
5	5 (10.4)
6	7 (14.6)

**Table:** 

Participants according to whether they did or did not address all their problems via optional modules	
	*N* (%)
Participants who addressed all their problems	6 (12.5)
Participants who dropped out before the 2nd session (in which the 1st optional module could be completed)	10 (20.8)
Participants who chose not to complete any optional module (but completed at least 2nd session)	2 (4.2)
Participants who dropped out before addressing all their problems, but who only completed matched optional modules	3 (6.2)
Participants who completed fewer optional modules than needed to address their problems, but who only completed matched optional modules	1 (2.1)
Participants who, instead of addressing all their problems by matched modules, completed at least one general module (and no unmatched module)	15 (31.2)
Participants who, instead of addressing all their problems by matched modules, completed at least one general module and one unmatched module	9 (18.8)
Participants who, instead of addressing all their problems by matched modules, completed at least one unmatched module (and no general module)	2 (4.2)
